# Patient-Level Pericoronary Adipose Tissue Mean Attenuation: Associations with Plaque Characteristics

**DOI:** 10.3390/jcdd11110360

**Published:** 2024-11-07

**Authors:** Katrine Schultz Overgaard, Thomas Rueskov Andersen, Roda Abdulkadir Mohamed, Sebastian Villesen Kristensen, Helle Precht, Jess Lambrechtsen, Søren Auscher, Kenneth Egstrup

**Affiliations:** 1 Cardiovascular Research Unit, Odense University Hospital Svendborg, 5700 Svendborg, Denmark; katrine.schultz.overgaard@rsyd.dk (K.S.O.);; 2 Department of Clinical Research, Faculty of Health Sciences, University of Southern Denmark, 5230 Odense M, Denmark; 3 Health Sciences Research Centre, UCL University College, 5230 Odense M, Denmark; 4 Institute of Regional Health Research, University of Southern Denmark, 5230 Odense M, Denmark; 5 Department of Radiology, Lillebaelt Hospital, University Hospitals of Southern Denmark, 6000 Kolding, Denmark; 6 Discipline of Medical Imaging and Radiation Therapy, University College Cork, T12 K8AF Cork, Ireland

**Keywords:** pericoronary adipose tissue (PCAT), perivascular adipose tissue (PVAT), inflammation, coronary CT angiography, coronary artery disease, plaque stabilization

## Abstract

Pericoronary adipose tissue attenuation (PCAT_a_), observed from coronary computed tomography angiography (CCTA), is emerging as an inflammation marker. This study evaluated the relationship between PCAT_a_ and plaque characteristics, including plaque type, burden, and coronary calcification. An observational study was conducted on 466 patients with suspected chronic coronary syndrome who underwent clinically indicated CCTA. PCAT_a_ was measured along the proximal 40 mm of the coronary arteries and averaged to represent the patient’s level. Plaque type was assessed, compositional plaque volumes were measured, and plaque burdens were quantified. The coronary calcification scores (CCSs) were categorized into groups. Statistical methods included *t*-tests, ANOVA, and multivariate regression analysis. PCAT_a_ differed significantly between calcified (−81.7 Hounsfield units (HU)) and soft (−77.5 HU) plaques. PCAT_a_ was positively associated with total plaque burden (β = 3.6) and non-calcified plaque burden (β = 7.0), but negatively correlated with calcified plaque burden (β = −3.5), independent of clinical factors and tube voltage (*p* < 0.05). The effect of PCAT_a_ was stronger when plaques of a different composition were absent. No significant differences in PCAT_a_ were found among different CCS groups. PCAT_a_ increased for calcified compared to soft plaques. The non-calcified plaque burden was associated with a higher PCAT_a_, while the calcified plaque burden was associated with a lower PCAT_a_.

## 1. Introduction

Coronary artery disease (CAD) is a leading cause of morbidity and mortality worldwide [1]. Inflammation is a key driver of atherogenesis, plaque progression, and rupture [2]. Plaque calcification displays dual roles, capable of exhibiting both pro- and anti-inflammatory characteristics [3]. However, the identification of coronary inflammation is challenging, as no existing biomarkers are suitable [4].

A novel approach, first described by Antonopoulos et al. [5], used coronary computed tomography angiography (CCTA) to measure the attenuation of the pericoronary adipose tissue (PCAT) as a marker of the ongoing inflammation. PCAT is characterized as the metabolic active adipose tissue located adjacent to the coronary arteries. PCAT can release pro-inflammatory cytokines and promote atherogenesis in response to inflammation [6]. It was recently revealed that the communication between the vascular wall and PCAT is bidirectional [7]. Inflammatory signals released from the inflamed vascular wall can induce a shift in the PCAT composition. On a CCTA, this will manifest as PCAT attenuation changing from a lipid-rich and highly negative value (from −190 Hounsfield unit (HU)) to a more aqueous, less negative value (approaching −30 HU) [8]. The clinical value of PCAT attenuation is currently under thorough investigation [9]. PCAT attenuation has been associated with CAD presence [5], plaque burden [5,10], and non-calcified plaque progression [10], and it can predict cardiac mortality [11]. However, since PCAT attenuation is a novel marker, variations in measurement methods exist, and significant differences are observed among the three coronary vessels. Gaps persist regarding whether PCAT attenuation can serve as a patient level marker, which could potentially enhance its clinical application.

Atherosclerosis is a complex process in which plaques can progress or regress, with inflammation playing a pivotal role. Plaque calcification can follow two paths based on macrophage polarization; M1 macrophages promote micro-calcification that is associated with plaque rupture, while M2 macrophages promote macro-calcification that stabilizes the plaque [3]. Gaps remain in understanding how PCAT attenuation is associated with plaque calcification.

In this observational study of patients suspected of exhibiting chronic coronary syndrome, we used CCTA to compare the association of patient-level proximal pericoronary adipose tissue attenuation with (i) the presence and type of coronary plaques, (ii) plaque volumes and burden, and (iii) coronary calcification.

## 2. Materials and Methods

### 2.1. Study Design

This was a single-center, prospective observational study of patients referred for a CCTA due to suspicion of chronic coronary syndrome. Patients underwent evaluation at the Outpatient Clinic of Cardiology at Odense University Hospital, Svendborg, between January 2018 and February 2020. The study adhered to the principles of the Declaration of Helsinki and received approval from the Regional Committees on Health Research Ethics for Southern Denmark (ID S-20170094) and the Danish Data Protection Agency (ID 18/5857). As this was a purely observational study with no medical intervention, it was not registered on ClinicalTrials.gov.

### 2.2. Study Population

**Figure 1:**
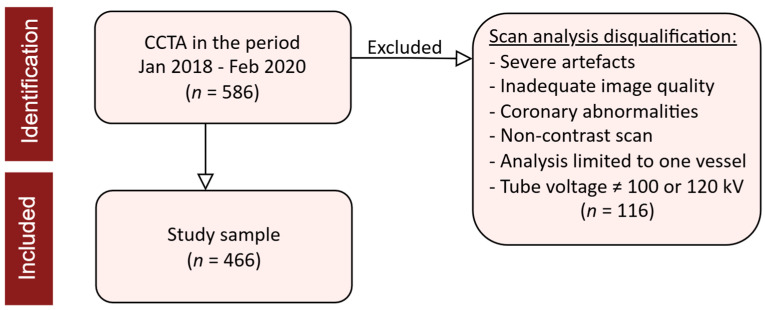
Flowchart. This flowchart illustrates the study sample selection, including the identification of patients and their eligibility based on exclusion criteria related to scan analysis. CCTA = coronary CT angiography; Jan = January; Feb = February; *n* = number; kV = kilovolt.

A total of 586 patients, who underwent CCTA for clinical indications and provided consent between January 2018 and February 2020, were consecutively included in the analysis (Figure 1). During the CCTA assessments, 116 scans were excluded due to severe artefacts, inadequate image quality, coronary abnormalities, non-contrast scans, analysis limited to only one vessel, or tube voltage other than 100 or 120 kilovolt (kV) (*n* = 53). Tube voltage is considered to affect PCAT attenuation, independent of patient size, as indicated by a study from Ma et al. [12], who found that only scans conducted at 100 and 120 kV were comparable. Similarly, Takagi et al. [13] noted changes in plaque composition with 80 kV, but no notable differences between 100 and 120 kV. Inclusion criteria for participation included (I) age ≥ 18 years, (II) ability to provide informed consent, and (III) referral for CCTA due to suspicion of chronic coronary syndrome. Exclusion criteria included (I) body mass index (BMI) > 40, (II) irregular or fast heart rhythm inadequate for CCTA, (III) reduced kidney function with estimated glomerular filtration rate (eGFR) <45 mL/min, (IV) contrast allergy, (V) inadequate image quality, and (VI) tube voltage < 100 or >120 kV.

Patients visited the facility for blood sample collection and the completion of questionnaires near the time of their CCTA. The mean interval between the CCTA and the blood sample collection was 29 days. Blood samples were analyzed for glycated hemoglobin A1c (HbA1c), total cholesterol, low-density lipoprotein (LDL), eGFR, and C-reactive protein (CRP). Cardiovascular risk factors were assessed through a questionnaire covering age, height, weight, smoking status, history of CAD, medical history, and medication use. Written and oral consent were obtained for each participant before the examination.

### 2.3. CCTA Acquisition

All patients underwent CCTA using a 256-slice scanner (GE Healthcare, Revolution, Milwaukee, WI, USA). Patients were administered a 7.5 mg tablet of ivabradine the evening before the CCTA and another 7.5 mg the morning of the CCTA procedure. Patients received sublingual nitroglycerin just before the scan. Those with a heart rate > 65 bpm before the scan received 5 mg doses of an intravenous beta-blocker, up to an accumulated maximum of 30 mg. Images were acquired using electrocardiogram-gated prospective acquisition at the 75% level of the R-R interval, with an additional padding of 45 ms for reconstruction. The tube voltage was adjusted based on the patient’s body size.

Initially, an unenhanced scan was performed to estimate the coronary calcium score (CCS), utilizing the scoring system described by Agatson et al. [14]. Following this, a contrast-enhanced coronary artery scan was performed to evaluate plaque presence and characteristics. Iodine contrast (Visipapque 320 mg/mL) was injected, with the dose and flow rate dependent upon patient characteristics. The timing of the scan was synchronized to match with maximal attenuation in the ascending aorta. The CCTA images were reconstructed at a slice thickness of 0.625 mm.

### 2.4. Image Analysis

The CCTA images were analyzed using the validated software program QAngioCT Research Edition, version 3.2.0.13 (Medis Medical Imaging, Leiden, The Netherlands) [15]. Experienced observers conducted the analysis blinded to patient characteristics. The software automatically generated the centerlines for each coronary vessel. Manual adjustments were made to the longitudinal and cross-sectional lumen and vessel contours, when necessary. Automated segmentation was performed by the software and manually corrected as needed, following the 16-segment coronary artery tree model [16].

All segments underwent visual examination to detect the presence of plaques, defined as structures ≥ 1 mm^3^ within or adjacent to the lumen and visible in ≥2 planes. Plaques were classified into three types based in regards to attenuation: calcified >80%, mixed 20–80%, and soft <20%. For statistical analysis, in patients with more than one plaque, the plaque exhibiting the most clinically significant type was used, prioritized as follows: soft, mixed, and calcified.

For each patient, the plaque volumes were determined by the automated calculation of the total plaque volume (PV) (mm^3^) based on fixed HU thresholds. The total PV was further sub classified into compositional PVs, using the following predefined HU cut-off values [17]: (1) non-calcified (−30 to −350 HU) PV, i.e., necrotic core (−30 to 30 HU) PV, fibro-fatty (31 to 130 HU) PV, and fibrous (131 to 350 HU) PV; (2) calcified PV (>351 HU).

The plaque burdens were quantified as the normalized atheroma volume (NAV) to adjust for differences in vessel length. NAV was defined as the total vessel volume minus the total lumen volume divided by the mean segment length. The total, non-calcified, and calcified plaque volumes were normalized using this formula.

### 2.5. PCAT Attenuation Measurement

**Figure 2:**
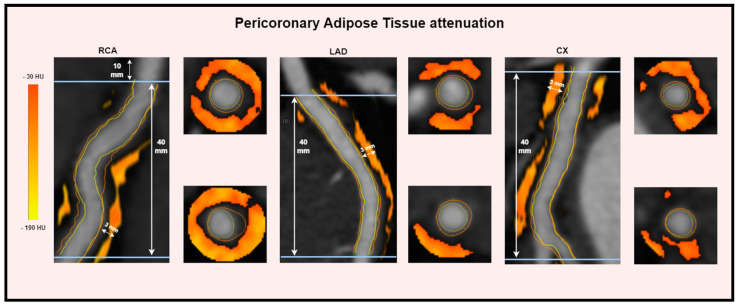
Method for quantifying pericoronary adipose tissue (PCAT) attenuation. PCAT attenuation was measured on the 40 mm proximal segments of the RCA (right coronary artery), the LAD (left anterior descending), and the CX (circumflex). Images from a patient case using QAngioCT Research Edition, version 3.2.0.13, Medis Medical Imaging, including color maps showing curved MPR (multiplanar reconstruction) and cross-sectional views at the starting and ending points of the segments.

PCAT attenuation was measured using the fully automated QAngioCT Research Edition, version 3.2.0.13 (Medis Medical Imaging, Leiden, The Netherlands). The PCAT measurements were conducted on the semi-automatically adjusted segmentations, as described above. PCAT attenuation was measured in all three major coronary arteries, i.e., the RCA (right coronary artery), LAD (left anterior descending), and CX (circumflex), and averaged to represent the patient level as the PCAT mean attenuation (PCAT_MA_). PCAT_MA_ measurements were based on the framework proposed by Antonopoulus et al. [5]. PCAT refers to the volume located between the vessel wall and an outer radial distance of 3 mm, quantified with an attenuation ranging between −190 and −30 HU, indicative of adipose tissue (Figure 2) [5,18]. PCAT attenuation was measured for the proximal 40 mm segments, after branching from the left main into the LAD and CX, while measurements for the RCA were performed at the 10th–50th mm from the ostium to minimize any potential influence from the aortic wall.

### 2.6. Statistics

Statistical analysis was conducted using Stata 17.0 software (StataCorp. 2019. College Station, TX, USA). Baseline data were presented using descriptive statistics, including numbers (*n*) and percentages (%), means with standard deviations (±SD), or medians with interquartile ranges (IQR), based on the data distribution. Normality testing was assessed graphically, and relevant transformations of data were applied, as appropriate, to improve normality. A paired *t*-test was employed to examine PCAT_MA_ across the three major coronary vessels, while an unpaired *t*-test was used to compare PCAT_MA_ and plaque presence. One-way analysis of variance (ANOVA) testing was applied to compare PCAT_MA_ among the categories of plaque types and CCS groups. Multiple linear regression analyses were performed to explore the associations of PCAT_MA_ with plaque presence, type, volumes, and burdens. Three linear regression models were applied separately to each of the above variables. The basic regression model 1 was unadjusted; the multivariate model 2 included adjustments for sex, age, BMI, LDL, CRP, smoking status, antihypertensive- and lipid-lowering medication, and tube voltage. The multivariate model 3 was performed on the plaque burdens and included the adjustments employed in model 2, plus the non-calcified plaque burden and the calcified plaque burden. Statistical significance was defined as a *p*-value < 0.05 for all outcomes, with 95% confidence intervals reported as well.

## 3. Results

### 3.1. Clinical Characteristics

A total of 466 patients referred for a CCTA due to suspected chronic coronary syndrome were included in the study (Table 1). The mean age was 61 years, with a nearly balanced sex distribution (56% male). A total of 40% of patients were on antihypertensive medication, 37% were taking statins, 9% had diabetes, and fewer than 5% had a history of acute myocardial infarction or prior cardiovascular revascularization.

### 3.2. CCTA Findings

PCAT attenuation was averaged across all three coronary vessels to represent the patient-level PCAT_MA_, with a mean value of −80.4 HU (±6.0) (Table 1). PCAT attenuation differed significantly between the three major coronary vessels (Figure S1); however, the patient-level PCAT_MA_ showed strong correlations across all vessels (Figure S2). Among the 466 patients, 275 (59%) exhibited one or more plaques in their coronary vessels. The predominant plaque type identified was mixed (44%), followed by solely calcified plaques (CP) (32%), and soft plaques (25%). The total PV across all three coronary vessels was 842 (±263) mm^3^, primarily consisting of non-calcified plaque (NCP) PV at 749 (±189) mm^3^, while the median calcified PV was 28 (IQR: 10–68) mm^3^. Within the NCP, the volumes comprised 545 (±178) mm^3^ fibrous PV, 164 (IQR: 116–235) mm^3^ fibro-fatty PV, and 15 (IQR: 7–28) mm^3^ necrotic core PV. The total plaque burden exhibited a median of 2.8 (IQR: 2.4–3.3) mm^2^, comprised of a 0.1 (IQR: 0.03–0.3) mm^2^ CP burden and a 2.5 (IQR: 2.2–3.0) mm^2^ NCP burden.

### 3.3. Association Between PCAT_MA_, Plaque Types, and CCS

**Figure 3:**
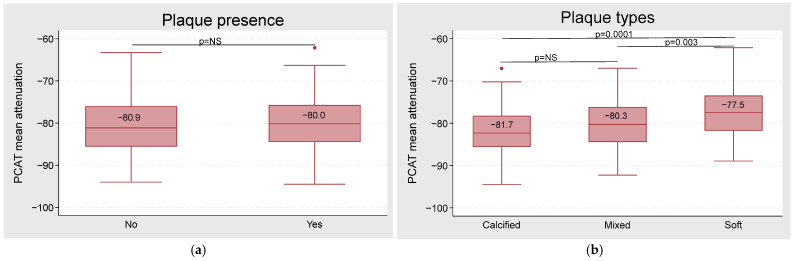
PCAT_MA_ by plaque presence and type. (**a**) Box plot of PCAT attenuation by plaque presence. (**b**) Box plot of PCAT attenuation by type of plaque. Dots represent outliers, indicating values that fall outside the expected range for each group. *p* < 0.05 was considered significant. PCAT = pericoronary adipose tissue.

No significant difference was observed in PCAT_MA_ regarding the presence of plaque (−80.0 ± 5.8 HU) or its absence (−80.9 ± 6.3 HU; *p* = 0.1) (Figure 3a, Table S1). However, the type of plaque significantly influenced PCAT_MA._ PCAT_MA_ increased from calcified (−81.7 HU) to soft plaques (−77.5 HU) and from mixed (−80.3 HU) to soft plaques (*p*-values < 0.05; Figure 3b). This association remained statistically significant from the calcified to the soft plaques in the multivariate-adjusted model 2 (Table 2). No significant differences in PCAT_MA_ levels were found across different stages of CCS categories (*p* > 0.5) (Figure S3, Table S2).

### 3.4. Association Between PCAT_MA_, Plaque Volumes, and Burden

In the basic model 1, PCAT_MA_ showed a positive association with total plaque volume (β coefficient (β) = 0.005 [0.003; 0.007]; *p* < 0.0001) and NCP volume (β = 0.01 [95% CI: 0.007; 0.01]; *p* < 0.0001), while PCAT_MA_ was negatively associated with calcified volume (β = −0.4 [95% CI: −0.8;−0.006]; *p* < 0.04)) (Table 2). The volumes of NCP included fibrous PV, fibro–fatty PV, and necrotic core PV, all of which exhibited a positive association with PCAT_MA_. After adjusting for CAD risk factors, biochemistry, medication, and tube voltage in multivariate model 2, this association persisted for the fibrous volume (β = 0.008 [95% CI: 0.005; 0.01]; *p* < 0.0001). Additionally, PCAT_MA_ demonstrated a positive association with total plaque burden (β = 4.6 [95% CI: 2.4; 6.8]; *p* < 0.0001) and NCP burden (β = 8.5 [95% CI: 5.9; 11.2]; *p* < 0.0001), while the association with CP burden was negative (β = −3.3 [95% CI: −5.9; −0.7]; *p* = 0.02). Adjustments for CAD risk factors and tube voltage in model 2 did not alter the associations with the different plaque burdens (*p* < 0.05). The multivariate model 3 also accounted for both CP and NCP burden. In this model, the positive association between PCAT_MA_ and NCP burden was reaffirmed (β = 9.1 [95% CI: 6.3; 12.0]; *p* < 0.0001), and a significant negative association between CP burden (β = −6.5 [95% CI: −9.4; −3.6]; *p* < 0.0001) was also confirmed.

## 4. Discussion

In this study, we investigated the relationship between patient-level proximal PCAT attenuation and plaque characteristics in patients suspected of exhibiting chronic coronary syndrome. Our findings, illustrated in the graphical abstract, revealed that (1) PCAT_MA_ increased progressively from calcified to soft plaques; (2) NCP volume and burden were associated with higher PCAT_MA_; (3) CP burden was associated with lower PCAT_MA_. The effects of CP burden and NCP burden were more pronounced when the opposite plaque type was absent.

### 4.1. Inflammation in Atherosclerosis

Inflammation plays a critical role in the development of atherosclerosis and is typically more pronounced in the early stages. NCP often emerge early in the atherosclerotic process, while CP tend to develop later [19]. Our study observed a gradual increase in PCAT_MA_ from calcified to soft plaques. These findings are consistent with those of other studies [20,21,22] that identified a similar progression in plaque vulnerability types. In a comprehensive CCTA study examining 3500 patients with symptomatic chronic coronary syndrome, the mortality rates were found to increase with plaque vulnerability type, i.e., 1.4% for CP, 3.3% for mixed plaque, and 9.6% for NCP [23], thus underscoring the clinical significance of the plaque type. We also found that NCP volume and burden were positively associated with PCAT_MA_. Other studies have similarly found significant associations between PCAT attenuation, as well as progression [10], of NCP burden [24].

### 4.2. Interpreting the Variability of PCAT Measurements

The initial validation of PCAT attenuation was limited to the 40 mm proximal segments of the RCA [5]. This raised concerns regarding the representativeness of PCAT attenuation measurements, especially after studies revealed notable variations between the three major coronary arteries [12]. In response, the same research group extended its investigation to also include the validation of LAD and CX [11]. This led to the hypothesis that the proximal segments of all three major coronary arteries could serve as indicators of the inflammatory load of the respective vessels.

In our study, we sought to extend this concept by evaluating PCAT attenuation at the patient level. We calculated the PCAT_MA_ across all three major coronary arteries, hypothesizing that this could represent the patient level. To our knowledge, our study is the first to associate patient-level PCAT_MA_ with increasing plaque vulnerability types, as well as NCP volume and burden. Similarly, Giesen et al. measured PCAT attenuation in each of the three coronary arteries and found signification associations with NCP burden [24]. Previous studies [20,21] have shown that lesion-specific PCAT attenuation, measured locally around the plaque, associates with plaque vulnerability types, with increasing values from calcified to soft plaques.

The method of measuring lesion-specific PCAT attenuation has raised concerns about its comparability. PCAT attenuation varies along the vessel length; it decreases from the proximal to the distal segments of the vessels [25]. The variation is attributed to the number of side branches, along with other less defined biological factors [9]. Furthermore, the inherent differences between the three major coronary arteries, combined with varying locations and lengths of plaques, further challenges the comparability of lesion-specific PCAT attenuation. Notably, when Ma et al. [20] compared lesion-specific PCAT attenuation with a proximal patient-level approach, they found that the lesion-specific PCAT attenuation correlated more strongly with plaque development and vulnerability than did the proximal patient-level approach. The discrepancies between our findings and those from Ma et al. may arise from measurement of different proximal segment lengths; we analyzed 40 mm segments, while Ma et al. restricted the measurements to 10 mm segments. Building upon the lesion-specific PCAT approach, another study [22] extended the measurements to include the 10 mm segments both proximal and distal to the lesions. They observed a similar gradual increase in PCAT attenuation from CP to NCP, and this was present in the proximal and distal areas around the lesions. These findings strengthen the notion that PCAT attenuation may be influenced on a more global scale.

### 4.3. The Role of Calcification in Coronary Inflammation

The relationship between calcification and coronary inflammation is important in understanding the natural history of atherosclerosis. A study by Jing et al. [26] found that PCAT attenuation was higher in patients with CAD and a CCS of zero compared to those with a CCS above zero. They suggested that the presence of calcium might stabilize the plaque and thereby reduce the inflammatory activity. Our finding that CP burden was negatively associated with PCAT attenuation adds to this understanding and supports the plaque stabilization hypothesis that calcification might stabilize the vessel and indirectly contribute to reduce inflammation. Furthermore, we found that the effects of CP burden on PCAT_MA_ were more pronounced when NCP was absent. Thus, both studies indicate that the impact of calcification on PCAT attenuation is affected by the presence of NCP.

Our study revealed no significant variations in PCAT_MA_ across different levels of CCS. This result agrees with studies in the existing literature that have shown no associations between CCS categories [11,20,26], CP burden [11], or progression of CP burden [10]. The lack of clear associations may be attributed to the inherent complexity of atherosclerotic disease processes, in which the simultaneous presence of CP and NCP in patients across different CCS categories might explain these findings. Atherosclerosis is a dynamic disease that can harbor multiple plaques at different development stages simultaneously. Our findings could suggest that the presence of NCP might play a more significant role than the mitigating effects of calcification.

The pathophysiological mechanism of calcification reveals the complexity of the processes. Plaque calcification displays dual roles, as it is can exhibit both pro- and anti-inflammatory characteristics. Macrophages play a key role. M1 phenotype macrophages are associated with the progression of atherosclerosis, facilitating micro-calcification, and potentially destabilizing plaques, while M2 phenotype macrophages are related to the regression phase, promoting macro-calcification and contributing to plaque stability [3].

PCAT attenuation shows promise as a biomarker capable of distinguishing inflammation in the atherosclerotic process. Although still speculative, it is hypothesized that patients with minimal plaque burden, or even no plaques, might exhibit elevated PCAT attenuation, indicative of the beginning of atherosclerosis. Conversely, in cases of widespread atherosclerosis, elevated PCAT attenuation may signify acute inflammation, while normal or reduced PCAT attenuation could indicate resolved inflammation, possibly in response to medical treatment or as an indirect consequence of calcification [26].

The JUPITER trial [27] demonstrated that statin therapy significantly reduced CRP levels, independent of LDL reduction, underscoring the anti-inflammatory effects of statins. The PARADIGM study [28] further supported this result by showing that statins were associated with an increase in calcified plaque volume, alongside reduced progression of non-calcified plaques, suggesting that statins promote plaque stabilization and reduce inflammation. Similarly, Goeller et al. [10] observed a trend of reduced PCAT attenuation among patients who started statin therapy a year after baseline CCTA compared to those who did not. Taken together, these findings could suggest that reductions in PCAT attenuation observed with statin therapy potentially reflect shifts toward a more stable, less inflammatory plaque environment.

### 4.4. Methodological Implications

Our study has some limitations that should be considered. Firstly, the observational design limits our ability to establish causality. To address this, prospective longitudinal studies or randomized controlled trials are recommended in the future. Secondly, the single-center design may not reflect broader populations adequately; also, our cohort consisted of participants with low CAD risk. Extending the research to multicenter collaborations could enhance generalizability. The sample size of our study was comparable to that employed in prior research within this field, which has identified significant effects under similar conditions. We employed a validated software program and observers experienced in the assessment of plaque and PCAT_MA_, which ensures robust data interpretation, but manual adjustments during image analysis might introduce variability. Technical factors regarding imaging methods and individual patient differences can affect the quantification of PCAT_MA_; some of these concerns have been addressed in the Section 4.2. Further research is needed to refine measurement techniques and explore biological mechanisms affecting PCAT, particularly regarding their relationship with calcification.

## 5. Conclusions

Our study reveals a nuanced interplay between patient-level pericoronary adipose tissue attenuation and plaque characteristics. PCAT_MA_ increased from calcified to soft plaques and with greater NCP volume and burden. Conversely, PCAT_MA_ decreased with greater CP burden. The effects of CP and NCP burden on PCAT_MA_ were more pronounced when the opposite plaque type was absent. Future research is essential to explore the biological mechanisms influencing PCAT attenuation.

## Figures and Tables

**Table 1:** Patients demographics and CCTA findings.

Demographic Data	*n* = 466
Age, years (SD)	61.2 (±10.7)
Sex, male (%)	263 (57)
BMI, kg/m^2^ (IQR)	27.8 (25.4–31.2)
Smoking, *n* (%)	
Active	67 (14)
Former	200 (43)
Never	199 (43)
Antihypertensive medication, *n* (%)	184 (41)
Statins, *n* (%)	170 (37)
Diabetes, *n* (%)	42 (9)
Prior AMI, *n* (%)	10 (2%)
CV revascularization, *n* (%)	15 (3%)
Family history of CAD, *n* (%)	159 (34)
**Laboratory data**	
eGFR, mL/min/1.73 m^2^ (IQR)	86 (76–90)
HbA1c, mmol/mol (IQR)	37 (35–40)
Cholesterol, mmol/L (SD)	4.8 (±1.1)
LDL, mmol/L (SD)	2.8 (±1.0)
CRP, mg/L (IQR)	1.6 (0.8–3.5)
**CCTA findings**	
Mean PCAT, HU (SD)	−80.4 (±6.0)
RCA-PCAT, HU (SD)	−81.3 (±7.5)
LAD-PCAT, HU (SD)	−82.8 (±6.2)
CX-PCAT, HU (SD)	−76.6 (±7.2)
Plaque present, *n* (%)	275 (59)
Type of plaque, *n* (%)	
Calcified	87 (32)
Mixed	120 (44)
Soft	68 (25)
Total CCS, (IQR)	21 (0–167)
CCS groups, *n* (%):	
0	124 (30)
1–99	158 (38)
100–399	77 (19)
>400	54 (13)
Missing CCS-data	53 (11)
Plaque volumes, mm^3^	
Total plaque, (SD)	842 (±263)
Calcified, (IQR)	28 (10–68)
Fibrous, (SD)	545 (±178)
Fibro–fatty, (IQR)	164 (116–235)
Necrotic core, (IQR)	15 (7–28)
Non-calcified, (SD)	749 (±189)
Plaque burden, mm^2^:	
Total NAV, (IQR)	2.8 (2.4–3.3)
Calcified NAV, (IQR)	0.1 (0.03–0.3)
Non-calcified NAV, (IQR)	2.5 (2.2–3.0)
Tube voltage, kV (%)	
100, *n* (%)	361 (77)
120, *n* (%)	105 (23)

Values are mean ± standard deviation (SD), median + interquartile range (IQR), or counts (*n*) + proportions (%). BMI = body mass index; CAD = coronary artery disease; eGFR = estimated glomerular filtration rate; HbA1c = glycated hemoglobin A1C; LDL = low-density lipoprotein; CRP = C-reactive protein; PCAT = pericoronary adipose tissue; HU = Hounsfield unit; RCA = right coronary artery; LAD = left anterior descending; CX = circumflex; CCS = coronary calcium score; NAV = normalized atheroma volume; kV = kilovolt.

**Table 2:** Multivariate linear regression models for PCAT_MA_ and plaque characteristics.

	Basic Model 1			Multivariate Model 2			Multivariate Model 3		
Variables	β	95% CI	*p*-Value	β	95% CI	*p*-Value	β	95% CI	*p*-Value
**Types of plaque:**									
Calcified (*n* = 87)	0 (ref)			0 (ref)					
Mixed (*n* = 120)	1.4	[−0.2; 3.0]	0.08	0.8	[−0.7; 2.3]	0.3			
Soft (*n* = 68)	4.2	[2.4; 6.0]	<0.0001	2.7	[0.9; 4.5]	0.004			
**Plaque volumes, mm^3^:**									
Total plaque	0.005	[0.003; 0.007]	<0.0001	0.004	[0.002; 0.006]	0.001			
Calcified ^a^	−0.4	[−0.8; −0.006]	0.047	−0.5	[−0.9; −0.02]	0.04			
Non-calcified	0.01	[0.007; 0.01]	<0.0001	0.008	[0.005; 0.01]	<0.0001			
Fibrous	0.006	[0.003; 0.009]	<0.0001	0.008	[0.005; 0.01]	<0.0001			
Fibro–fatty ^b^	3.1	[2.1; 4.1]	<0.0001	0.8	[−0.4; 2.0]	0.2			
Necrotic core ^b^	0.9	[0.3; 1.4]	0.002	−0.4	[−1.0; 0.2]	0.2			
**Plaque burden, NAV:**									
Total plaque burden ^b^	4.6	[2.4; 6.8]	<0.0001	3.6	[1.1; 6.0]	0.004	5.5	[−1.1; 12.1]	0.1
CP burden ^a^	−3.3	[−5.9; −0.7]	0.01	−3.5	[−6.4; −0.7]	0.02	−6.5	[−9.4; −3.6]	<0.0001
NCP burden ^b^	8.5	[5.9; 11.2]	<0.0001	7.0	[4.3; 9.8]	<0.0001	9.1	[6.3; 12.0]	<0.0001

Model 2 was adjusted for sex, age, BMI, smoking status, LDL, CRP, diabetes, statins, anti-hypertensive medication, and tube voltage. Model 3 included the adjustments for model 2, plus corrections for non-calcified plaque burden and calcified plaque burden. BMI = body mass index; LDL = low-density lipoprotein; CRP = C-reactive protein; PCAT = pericoronary adipose tissue; CI = confidence interval; NCP = non-calcified plaque; CP = calcified plaque; NAV = normalized atheroma volume. ^a^ Cube root transformed. ^b^ Log transformed.

## Data Availability

The data presented in this study are available on request from the corresponding author.

## Supplementary Materials

The following supporting information can be downloaded at: https://www.mdpi.com/article/10.3390/jcdd11110360/s1, Figure S1: PCAT by coronary vessels; Figure S2: Correlation of PCAT_MA_ and individual coronary vessels; Table S1: PCAT_MA_ by plaque presence; Figure S3: PCAT_MA_ by CCS groups; Table S2: PCAT_MA_ by CCS groups.

## References

[1] WHO (2024). The Top 10 Causes of Death.

[2] Ross R. (1999). Atherosclerosis—An inflammatory disease. N. Engl. J. Med..

[3] Shioi A., Ikari Y. (2018). Plaque Calcification During Atherosclerosis Progression and Regression. J. Atheroscler. Thromb..

[4] Kumric M., Borovac J.A., Martinovic D., Ticinovic Kurir T., Bozic J. (2021). Circulating Biomarkers Reflecting Destabilization Mechanisms of Coronary Artery Plaques: Are We Looking for the Impossible?. Biomolecules.

[5] Antonopoulos A.S., Sanna F., Sabharwal N., Thomas S., Oikonomou E.K., Herdman L., Margaritis M., Shirodaria C., Kampoli A.M., Akoumianakis I. (2017). Detecting human coronary inflammation by imaging perivascular fat. Sci. Transl. Med..

[6] Britton K.A., Fox C.S. (2011). Perivascular adipose tissue and vascular disease. Clin. Lipidol..

[7] Margaritis M., Antonopoulos A.S., Digby J., Lee R., Reilly S., Coutinho P., Shirodaria C., Sayeed R., Petrou M., De Silva R. (2013). Interactions between vascular wall and perivascular adipose tissue reveal novel roles for adiponectin in the regulation of endothelial nitric oxide synthase function in human vessels. Circulation.

[8] Antoniades C., Antonopoulos A.S., Deanfield J. (2020). Imaging residual inflammatory cardiovascular risk. Eur. Heart J..

[9] Antoniades C., Tousoulis D., Vavlukis M., Fleming I., Duncker D.J., Eringa E., Manfrini O., Antonopoulos A.S., Oikonomou E., Padró T. (2023). Perivascular adipose tissue as a source of therapeutic targets and clinical biomarkers: A clinical consensus statement from the European Society of Cardiology Working Group on Coronary Pathophysiology and Micro-circulation. Eur. Heart J..

[10] Goeller M., Tamarappoo B.K., Kwan A.C., Cadet S., Commandeur F., Razipour A., Slomka P.J., Gransar H., Chen X., Otaki Y. (2019). Relationship between changes in pericoronary adipose tissue attenuation and coronary plaque burden quantified from coronary computed tomography angiography. Eur. Heart J. Cardiovasc. Imaging.

[11] Oikonomou E.K., Marwan M., Desai M.Y., Mancio J., Alashi A., Hutt Centeno E., Thomas S., Herdman L., Kotanidis C.P., Thomas K.E. (2018). Non-invasive detection of coronary inflammation using computed tomography and prediction of residual cardiovascular risk (the CRISP CT study): A post-hoc analysis of prospective outcome data. Lancet.

[12] Ma R., Ties D., van Assen M., Pelgrim G.J., Sidorenkov G., van Ooijen P.M.A., van der Harst P., van Dijk R., Vliegenthart R. (2020). Towards reference values of pericoronary adipose tissue attenuation: Impact of coronary artery and tube voltage in coronary computed tomography angiography. Eur. Radiol..

[13] Takagi H., Leipsic J.A., Indraratna P., Gulsin G., Khasanova E., Tzimas G., Lin F.Y., Shaw L.J., Lee S.E., Andreini D. (2021). Association of Tube Voltage with Plaque Composition on Coronary CT Angiography: Results From PARADIGM Registry. JACC Cardiovasc. Imaging.

[14] Agatston A.S., Janowitz W.R., Hildner F.J., Zusmer N.R., Viamonte M., Detrano R. (1990). Quantification of coronary artery calcium using ultrafast computed tomography. J. Am. Coll. Cardiol..

[15] de Graaf M.A., Broersen A., Kitslaar P.H., Roos C.J., Dijkstra J., Lelieveldt B.P., Jukema J.W., Schalij M.J., Delgado V., Bax J.J. (2013). Automatic quantification and characterization of coronary atherosclerosis with computed tomography coronary angiography: Cross-correlation with intravascular ultrasound virtual histology. Int. J. Cardiovasc. Imaging.

[16] Austen W.G., Edwards J.E., Frye R.L., Gensini G.G., Gott V.L., Griffith L.S., McGoon D.C., Murphy M.L., Roe B.B. (1975). A reporting system on patients evaluated for coronary artery disease. Report of the Ad Hoc Committee for Grading of Coronary Artery Disease, Council on Cardiovascular Surgery, American Heart Association. Circulation.

[17] Chang H.J., Lin F.Y., Lee S.E., Andreini D., Bax J., Cademartiri F., Chinnaiyan K., Chow B.J.W., Conte E., Cury R.C. (2018). Coronary Atherosclerotic Precursors of Acute Coronary Syndromes. J. Am. Coll. Cardiol..

[18] Sjöström L., Kvist H., Cederblad A., Tylén U. (1986). Determination of total adipose tissue and body fat in women by computed tomography, 40K, and tritium. Am. J. Physiol..

[19] Joshi N.V., Vesey A.T., Williams M.C., Shah A.S., Calvert P.A., Craighead F.H., Yeoh S.E., Wallace W., Salter D., Fletcher A.M. (2014). ^18^F-fluoride positron emission tomography for identification of ruptured and high-risk coronary atherosclerotic plaques: A prospective clinical trial. Lancet.

[20] Ma R., van Assen M., Ties D., Pelgrim G.J., van Dijk R., Sidorenkov G., van Ooijen P.M.A., van der Harst P., Vliegenthart R. (2021). Focal pericoronary adipose tissue attenuation is related to plaque presence, plaque type, and stenosis severity in coronary CTA. Eur. Radiol..

[21] Jing M., Sun J., Zhou Q., Sun J., Li X., Xi H., Zhang B., Lin X., Deng L., Han T. (2023). Pericoronary adipose tissue differences among plaque types: A retrospective assessment. Clin. Imaging.

[22] Jing M., Xi H., Zhu H., Zhang B., Deng L., Han T., Zhang Y., Zhou J. (2023). Correlation of pericoronary adipose tissue CT attenuation values of plaques and periplaques with plaque characteristics. Clin. Radiol..

[23] Ahmadi N., Nabavi V., Hajsadeghi F., Flores F., French W.J., Mao S.S., Shavelle D., Ebrahimi R., Budoff M. (2011). Mortality incidence of patients with non-obstructive coronary artery disease diagnosed by computed tomography angiography. Am. J. Cardiol..

[24] Giesen A., Mouselimis D., Weichsel L., Giannopoulos A.A., Schmermund A., Nunninger M., Schuetz M., André F., Frey N., Korosoglou G. (2023). Pericoronary adipose tissue attenuation is associated with non-calcified plaque burden in patients with chronic coronary syndromes. J. Cardiovasc. Comput. Tomogr..

[25] Bao W., Chen C., Yang M., Qin L., Xu Z., Yan F., Yang W. (2022). A preliminary coronary computed tomography angiography-based study of perivascular fat attenuation index: Relation with epicardial adipose tissue and its distribution over the entire coronary vasculature. Eur. Radiol..

[26] Jing M., Xi H., Zhu H., Zhang X., Xu Z., Wu S., Sun J., Deng L., Han T., Zhang B. (2023). Is there an association between coronary artery inflammation and coronary atherosclerotic burden?. Quant. Imaging Med. Surg..

[27] Ridker P.M., Danielson E., Fonseca F.A.H., Genest J., Gotto A.M., Kastelein J.J.P., Koenig W., Libby P., Lorenzatti A.J., MacFadyen J.G. (2008). Rosuvastatin to Prevent Vascular Events in Men and Women with Elevated C-Reactive Protein. N. Engl. J. Med..

[28] Lee S.E., Chang H.J., Sung J.M., Park H.B., Heo R., Rizvi A., Lin F.Y., Kumar A., Hadamitzky M., Kim Y.J. (2018). Effects of Statins on Coronary Atherosclerotic Plaques: The PARADIGM Study. JACC Cardiovasc. Imaging.

