# Supplementary material for: Patient-Level Pericoronary Adipose Tissue Mean Attenuation: Associations with Plaque Characteristics
Source: J Cardiovasc Dev Dis. 2024 Nov 7;11(11):360. doi: 10.3390/jcdd11110360 (PMC11594772; doi:10.3390/jcdd11110360)
Supplement: Supplementary file 1 [file jcdd-11-00360-s001.zip › jcdd-3280473-supplementary.pdf]

Appendix

Supplemental Figure S1) Pericoronary adipose tissue (PCAT) attenuation (HU, Hounsfield units) values in the right coronary artery (RCA-PCAT), left anterior descending (LAD- PCAT) and left circumflex (CX- PCAT) coronary arteries. Dependent t-test between vessels**.**

S1 PCAT by coronary vessels

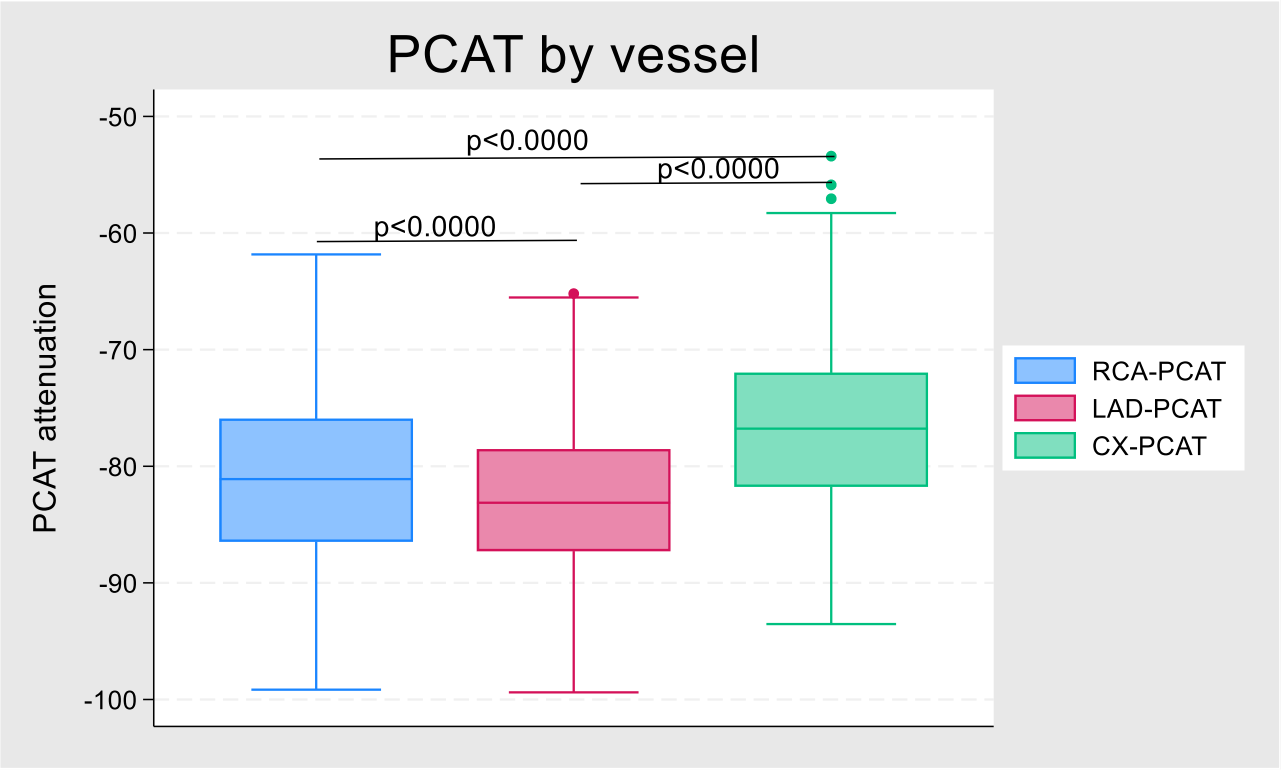

PCAT= Pericoronary adipose tissue, RCA= Right Coronary Artery, LAD= Left Anterior Descending, CX= Circumflex.

Supplemental Figure S2) Correlation and scatterplot with fit-line between PCAT mean attenuation ($PCAT_{MA}$) and RCA-PCAT, LAD-PCAT, and CX-PCAT, respectively.

S2) Correlation of $PCAT_{MA}$ and individual coronary vessels

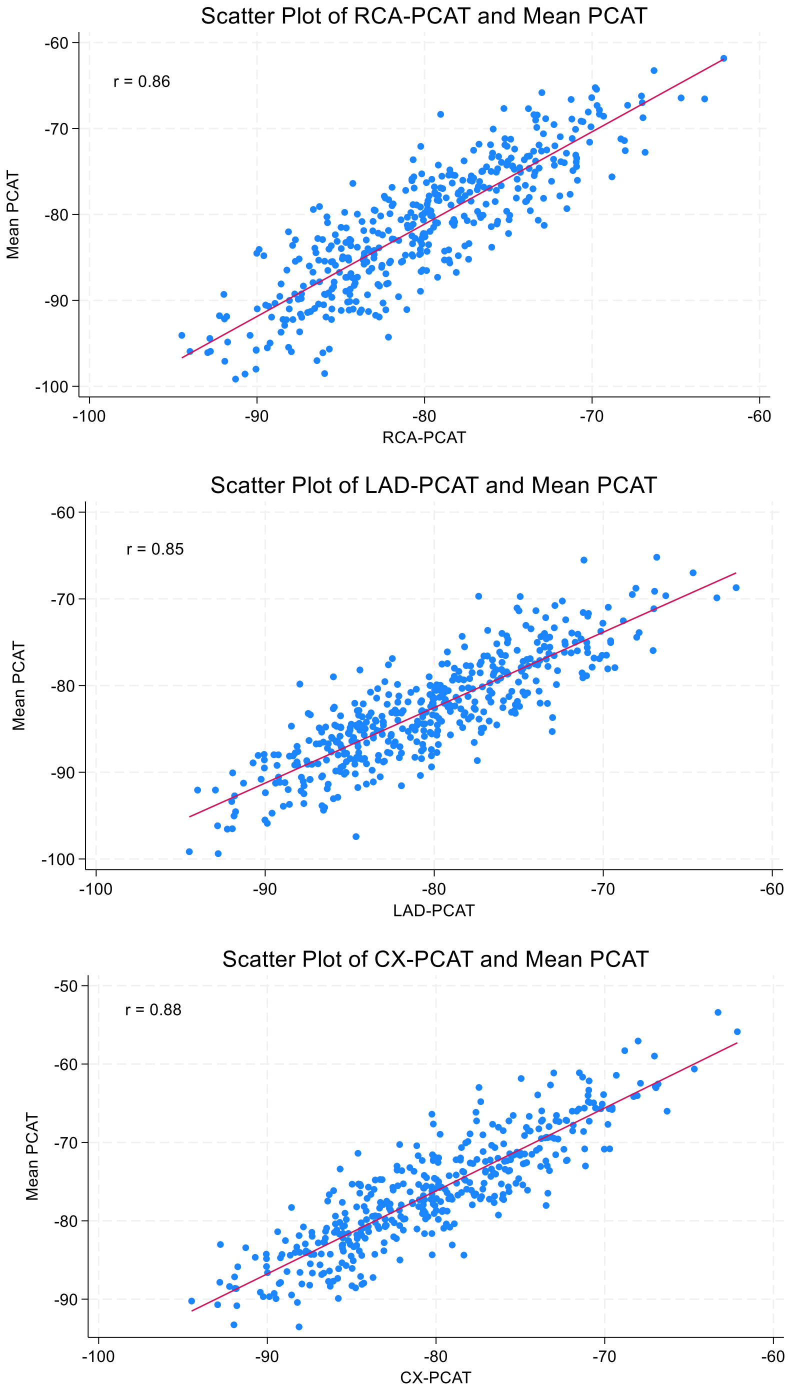

PCAT= Pericoronary adipose tissue, RCA= Right Coronary Artery, LAD= Left Anterior Descending, CX= Circumflex.

Table S1 $PCAT_{MA}$ by plaque presence

Plaque presence	$PCAT_{MA}$	±SD	95% CI
No	- 80.9	±6.3 HU	[-81.8;-80.0]
Yes	- 80.0	±5.8 HU	[-80.7;-79.3]

$PCAT_{MA}$= Pericoronary adipose tissue mean attenuation.

Figure S3 $PCAT_{MA}$ by CCS-groups

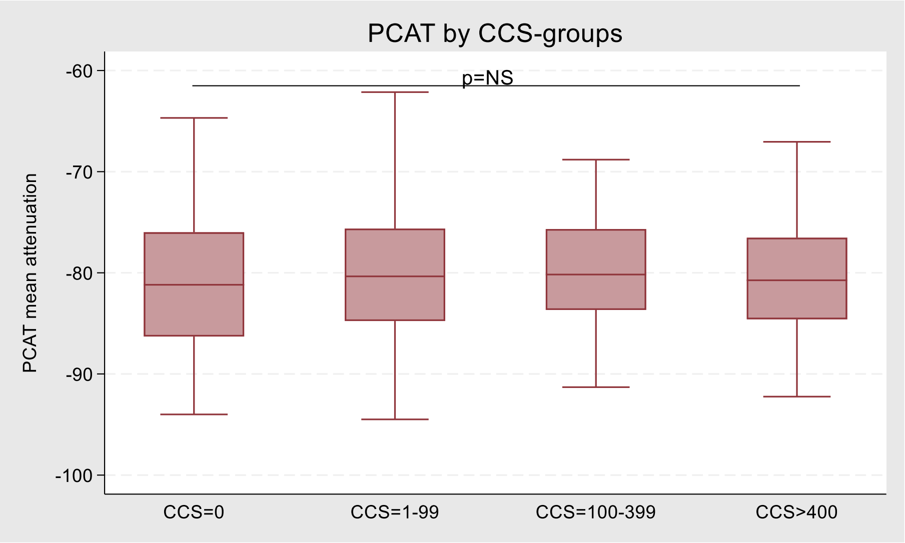

$PCAT_{MA}$= Pericoronary adipose tissue mean attenuation CCS= Coronary Calcium Score

Table S2 $PCAT_{MA}$ by CCS-groups

**CCS Groups:**	**0**	**1-99**	**100-399**
**0**	…	…	…
**1-99**	1.08 (p=0.8)	…	…
**100-399**	1.1 (p=1.0)	-0.01 (p=1.0)	…
**>400**	0.5 (p=1.0)	-0.6 (p=1.0)	-0.5 (p=1.0)

One-way analysis of variance (ANOVA) testing. PCAT= Pericoronary adipose tissue,

CCS= Coronary Calcium Score
